# Challenging the Skin Barrier: Intermediate-Term Success of Cryoablation for Superficial Invasive Mucinous Breast Cancer

**DOI:** 10.7759/cureus.95014

**Published:** 2025-10-20

**Authors:** Karen Fernandes, Haiyuan Shi, Yert Li Melissa Seet

**Affiliations:** 1 Radiology, Changi General Hospital, Singapore, SGP; 2 Breast Surgery, Changi General Hospital, Singapore, SGP

**Keywords:** breast cancer in an elderly patient, breast ultrasound, cryoablation, hydrodissection, minimally invasive, percutaneous cryoablation

## Abstract

We present a case of an 88-year-old woman with significant comorbidities, diagnosed with a 2.2 cm invasive mucinous breast carcinoma located directly beneath the skin without an intervening fat plane. Given her high surgical risk, the patient underwent percutaneous cryoablation instead of wide excision surgery. Real-time ultrasound-guided hydrodissection was used to create a protective plane between the tumor and dermis, displacing the lesion 10 mm away from the skin. Controlled freeze-thaw cycles were performed using a 13G IceCure probe, ensuring complete ablation while minimizing skin injury. The procedure was well tolerated with no complications. Serial follow-up imaging for three years demonstrated a progressive reduction in lesion size, evolving into a subcentimeter scar-like region. The patient remained asymptomatic with no recurrence. This case underscores cryoablation as a viable, minimally invasive alternative to surgery, even for patients with challenging tumor locations, advocating for broader adoption in selected elderly or high-risk patients.

## Introduction

Image-guided ablation techniques are being increasingly considered as non-surgical treatment options for early-stage breast cancer, especially for patients who are elderly, medically unfit for surgery, or in situations where curative surgery is not feasible [1-3]. Among these modalities, including radiofrequency ablation (RFA), laser ablation (LA), and microwave ablation (MWA), cryoablation shows particular advantage due to its precision, lower complication rates, and suitability for outpatient settings [4,5]. Cryoablation, delivered under local anesthesia using real‑time image guidance, offers precise tumor targeting, excellent cosmetic preservation, and high patient tolerance [3,6]. The ICE3 prospective trial reported a 96.3% ipsilateral breast tumor recurrence‑free rate at five years in low-risk patients aged ≥60 years. Breast cancer-specific survival was 96.7%, and both patients and physicians reported 100% satisfaction with cosmetic results, with no serious device-related adverse events [7]. Cryoablation is also emerging as a viable minimally invasive alternative for small, early-stage ER+/HER2− breast cancers, including mucinous subtypes, with favorable oncologic outcomes. Meta-analyses and follow-up studies report low recurrence and residual tumor rates in well-selected patients [4,5,8].

Despite these encouraging outcomes, superficial breast tumors abutting the dermis are commonly excluded from cryoablation due to concerns about thermal injury, skin necrosis and incomplete ablation [9,10]. Invasive mucinous carcinoma is a rare, indolent breast carcinoma subtype (1%-7% incidence), typically hormone receptor-positive and associated with favorable prognosis [11]. While surgical excision is standard management, select cases may benefit from non‑surgical management given the tumor's biology and patient condition. Our case report presents the intermediate‑term success of ultrasound‑guided cryoablation in an elderly patient with invasive mucinous carcinoma directly abutting the skin. By using continuous real‑time hydrodissection, we created a safety margin between the tumor and the dermis, enabling complete ablation despite the lesion’s superficial location.

## Case presentation

An 88-year-old Chinese woman presented with a painless lump in her left breast that she had noticed for several weeks. She denied nipple discharge and constitutional symptoms such as loss of appetite or recent weight loss. Her BMI was 24 kg/m^2^. Her medical history was notable for moderate-to-severe degenerative aortic stenosis, hypertension, dyslipidemia, asthma, gallstones, hepatitis B seroconversion, and recurrent COVID-19 infections. She experienced menarche at 16 and menopause at 53, had children, but did not breastfeed. She had used oral contraceptive pills for three years in her youth and had no history of hormonal replacement therapy. She was a non-smoker and non-drinker. She had no family history of breast or ovarian cancer. She had no history of breast surgery or trauma. She was a homemaker and remained cognitively intact, attending clinic visits with her son.

On inspection, both breasts were symmetrical with no visible masses, skin changes, or nipple abnormalities. On palpation, a 1.3-1.5 cm lobulated, mobile, non-tender nodule was felt at the 5 o’clock areolar margin of the left breast. No other lumps were detected in either breast, and there was no nipple discharge. There was no axillary, supraclavicular, or infraclavicular lymphadenopathy, and the overlying skin was intact.

**Figure 1:**
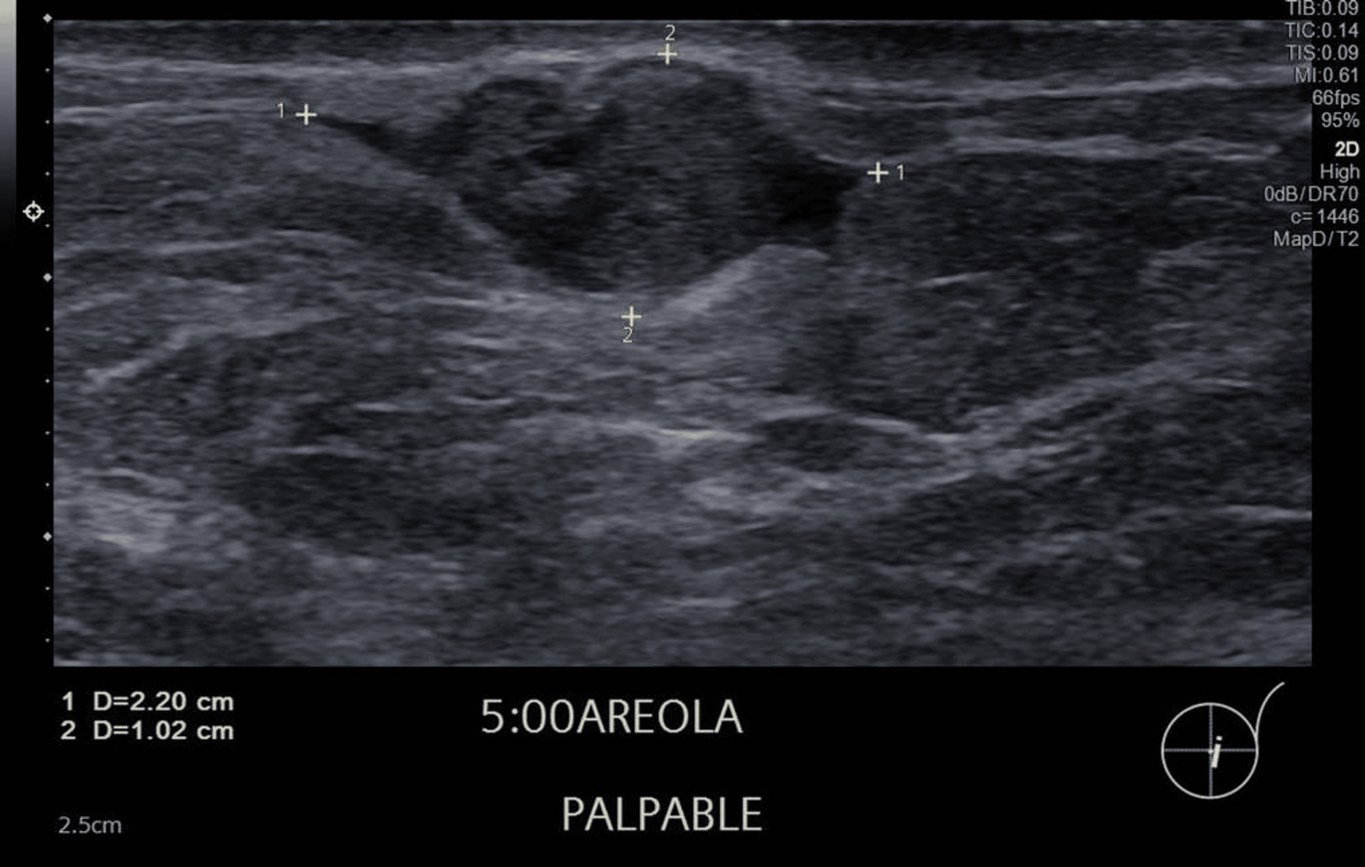
Left breast lesion under the skin Ultrasound showed a lobulated hypoechoic heterogenous nodule with small cystic areas, at 5 o'clock subareolar region under the skin in the left breast measuring 2.2 cm.

**Figure 2:**
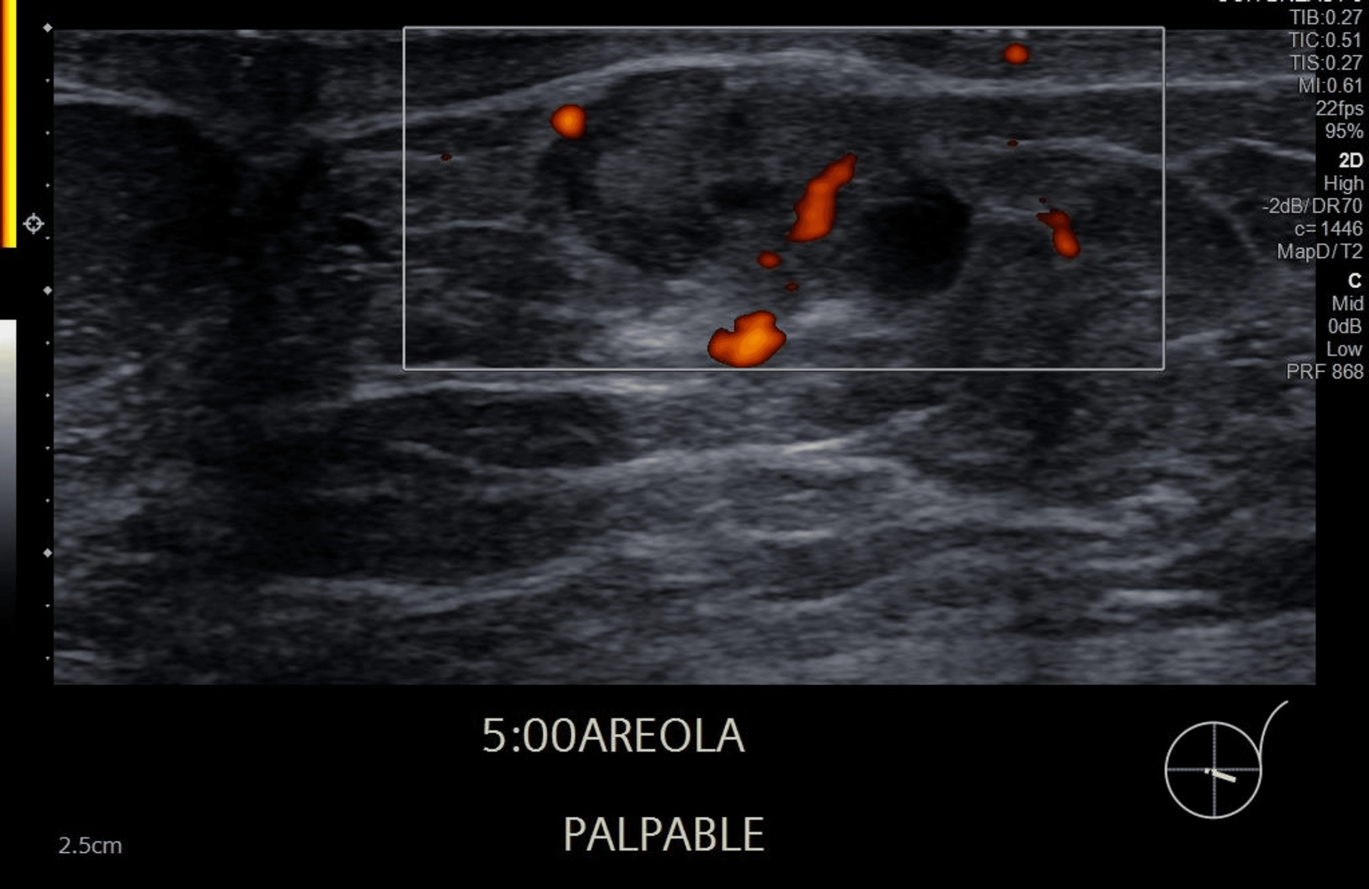
Intralesional vascularity Ultrasound revealed a lobulated, hypoechoic, heterogeneous nodule with small cystic areas and intralesional vascularity, located at the 5 o’clock subareolar region of the left breast, measuring 2.2 cm. Ultrasound-guided core biopsy confirmed invasive mucinous carcinoma (ER/PR positive, HER2 negative).

Initial ultrasound demonstrated a 2.2 cm lobulated hypoechoic nodule with internal cystic areas and intralesional vascularity, located directly beneath the dermis without an intervening fat plane (Figures 1, 2). No suspicious axillary lymphadenopathy was seen on ultrasound. Mammographic findings demonstrated a subareolar nodular density in a predominantly fatty breast, with no associated microcalcifications or architectural distortion. Further imaging for staging with CT and bone scan excluded regional or distant metastases. The formal clinical stage was cT2 cN0 cM0, corresponding to Stage IIA according to the American Joint Committee on Cancer (AJCC) 8th edition anatomic staging system.

A core needle biopsy of the lesion revealed invasive mucinous carcinoma. Immunohistochemistry demonstrated strong positivity for estrogen and progesterone receptors (both 100%, 3+), and HER2 was negative. Given the tumor’s biological profile and early-stage nature, the patient was started on endocrine therapy with tamoxifen. Due to her significant cardiac comorbidities and past COVID-19 reinfections, she was deemed to be high risk for general anesthesia. Despite initially agreeing to surgery, she and her family later declined operative management after cardiac review, citing safety concerns and the patient's frailty. She was also not keen to pursue transcatheter aortic valve intervention, which could have reduced her surgical risk. In addition, she declined axillary staging. The patient’s overall perioperative risk was classified as the American Society of Anesthesiologists (ASA) III.

**Figure 3:**
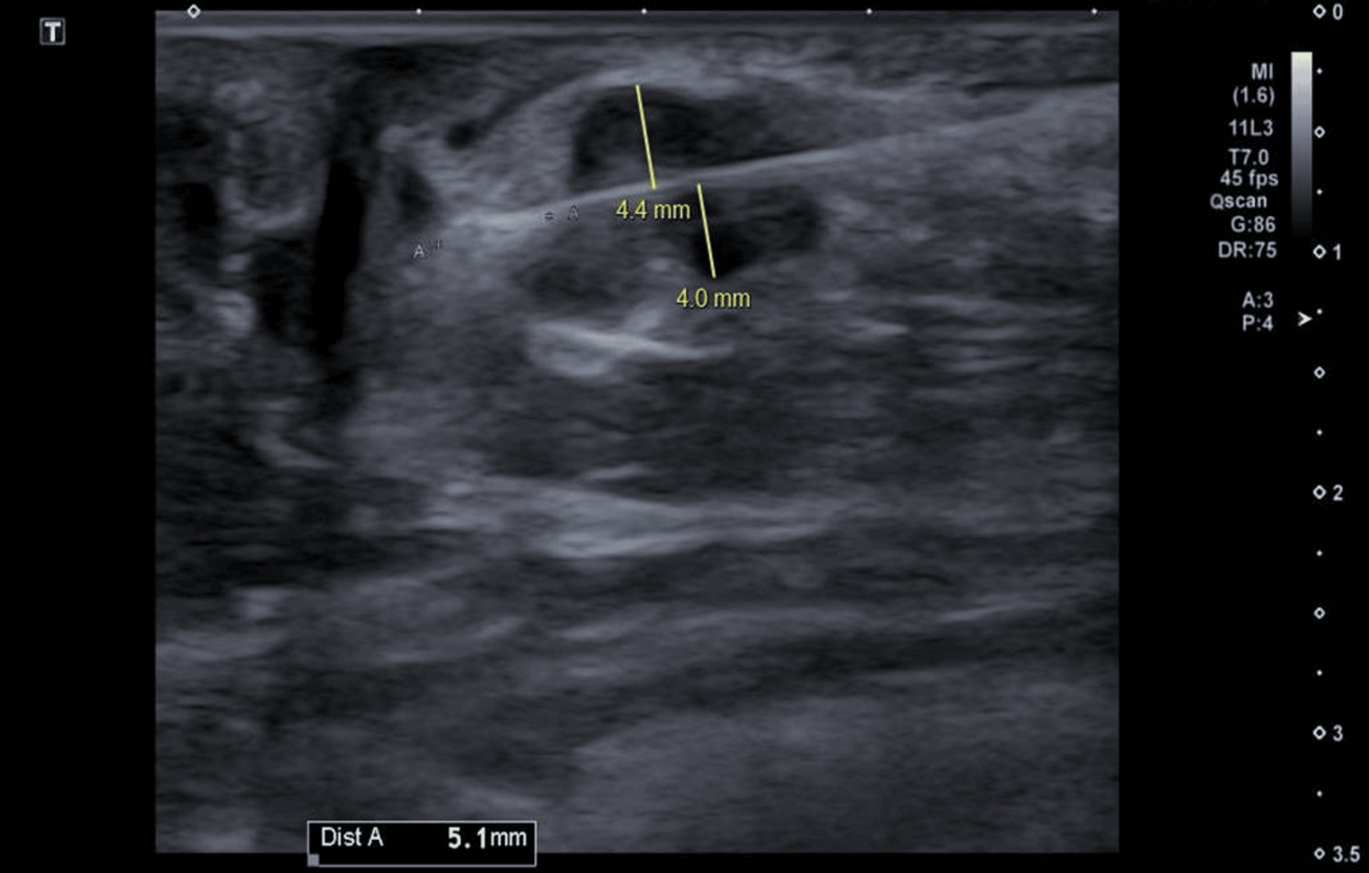
Skewering the Tumor During the cryoablation procedure, a 2.4 cm 13G IceCure probe was used to skewer the tumor along its longitudinal plane in a lateral-to-medial trajectory, with the probe tip extending 5 mm beyond the tumor margin.

**Figure 4:**
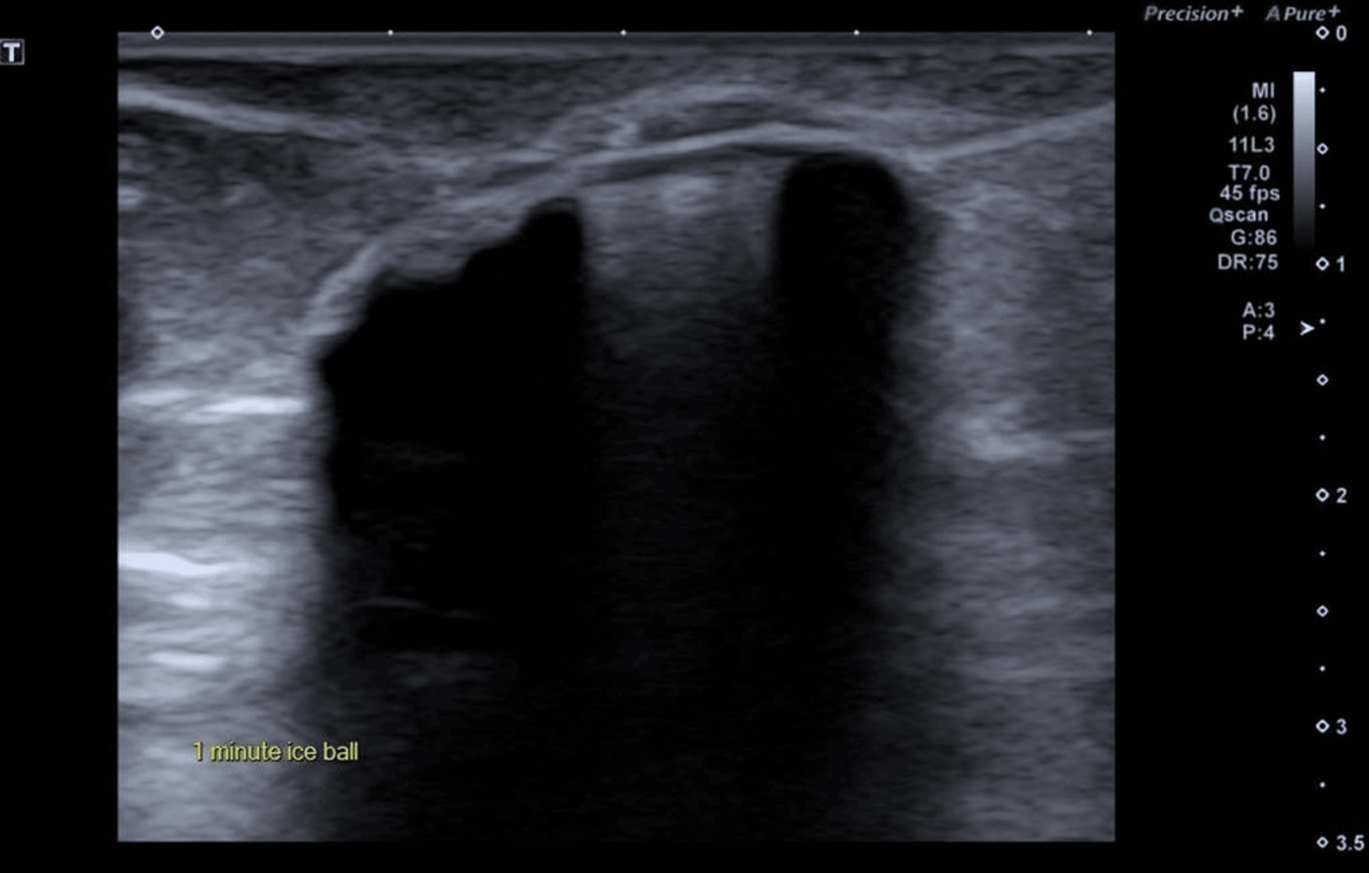
Creation of iceball Two cryoablation cycles were performed, resulting in a final iceball size of approximately 3.7x2.3 cm, corresponding to a lethal zone of about 3.2x2.0 cm.

**Figure 5:**
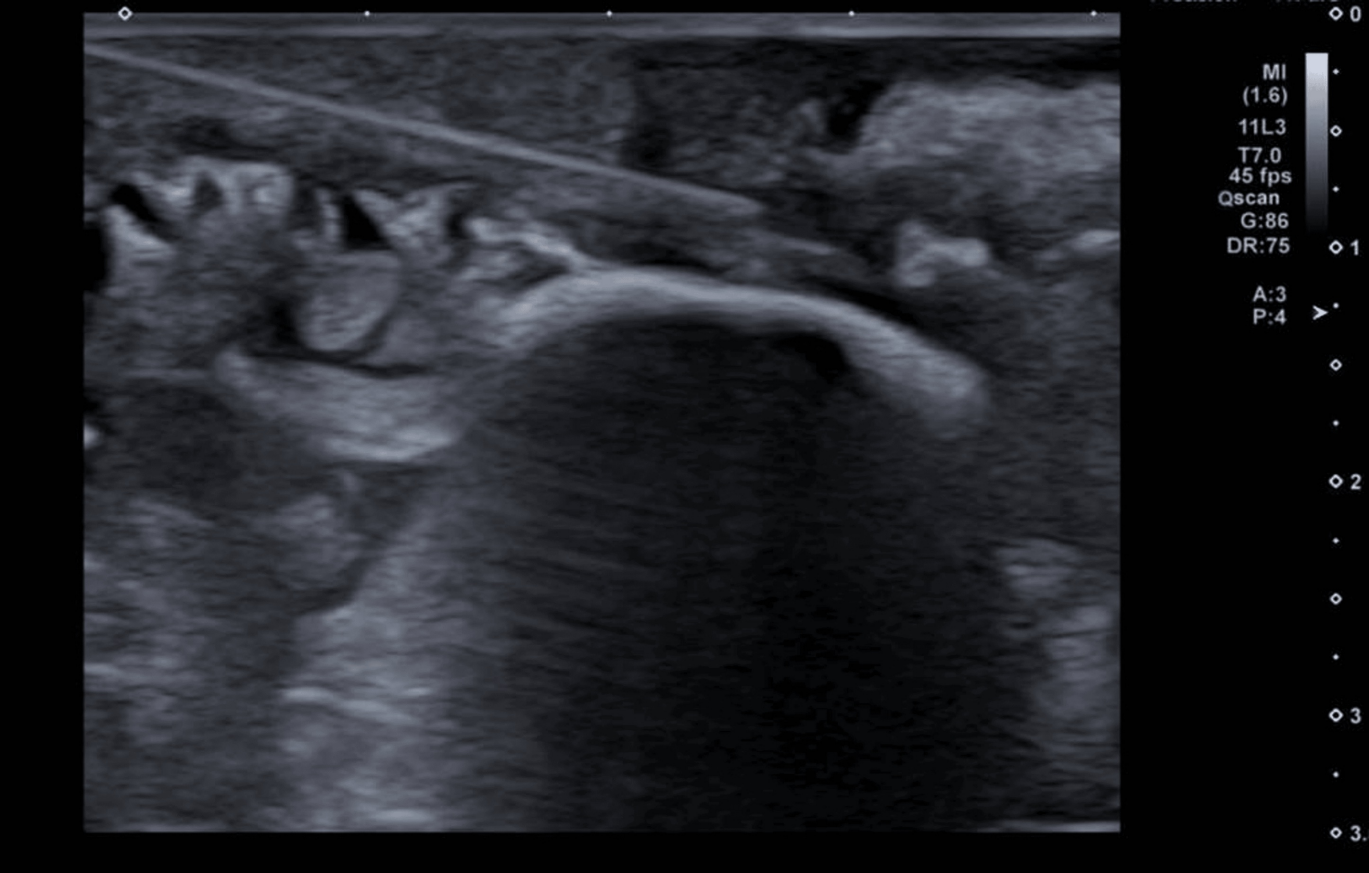
Hydrodissection to create a protective plane Continuous hydrodissection was performed using an 18G needle, displacing the tumor more than 10 mm from the skin to create a protective plane throughout the procedure.

After multidisciplinary discussion and shared decision-making, she was referred for percutaneous cryoablation as a minimally invasive alternative. Under local anesthesia and real-time ultrasound guidance, cryoablation was performed with meticulous technical planning. Large-volume continuous hydrodissection was achieved using 500 mL of normal saline mixed with 20 mL of 8.4% sodium bicarbonate, injected via an 18G needle to displace the tumor more than 10 mm away from the dermis, creating a safe margin. A 2.4 cm 13G IceCure cryoprobe (ProSense® system; IceCure Medical Ltd., Caesarea, Israel) was inserted in a lateral-to-medial direction along the lesion’s long axis, with the probe tip extending 5 mm beyond the tumor. The tumor was subjected to two controlled freeze-thaw cycles (four-minute freeze, 10-minute passive thaw, followed by a five-minute freeze), forming an iceball measuring approximately 3.7×2.3 cm, corresponding to a lethal ablation zone of about 3.2×2.0 cm (Figures 3-5). There were no immediate complications, and the patient tolerated the procedure well. Post-procedural care included continuation of endocrine therapy and routine surveillance.

The patient underwent cryoablation as a day procedure under local anesthesia and was discharged from the clinic on the same day after a short period of observation. She reported only minimal discomfort at the probe entry site, which was well controlled with simple oral analgesia. No immediate complications were observed.

The post-procedure plan included continuation of her prescribed endocrine therapy (tamoxifen), regular wound care advice, and scheduled imaging surveillance. She was reviewed at routine intervals in the breast clinic, with ultrasound and mammography performed at six months, one year, and yearly thereafter. Throughout follow-up, she remained clinically well and did not experience procedure-related morbidity.

**Figure 6:**
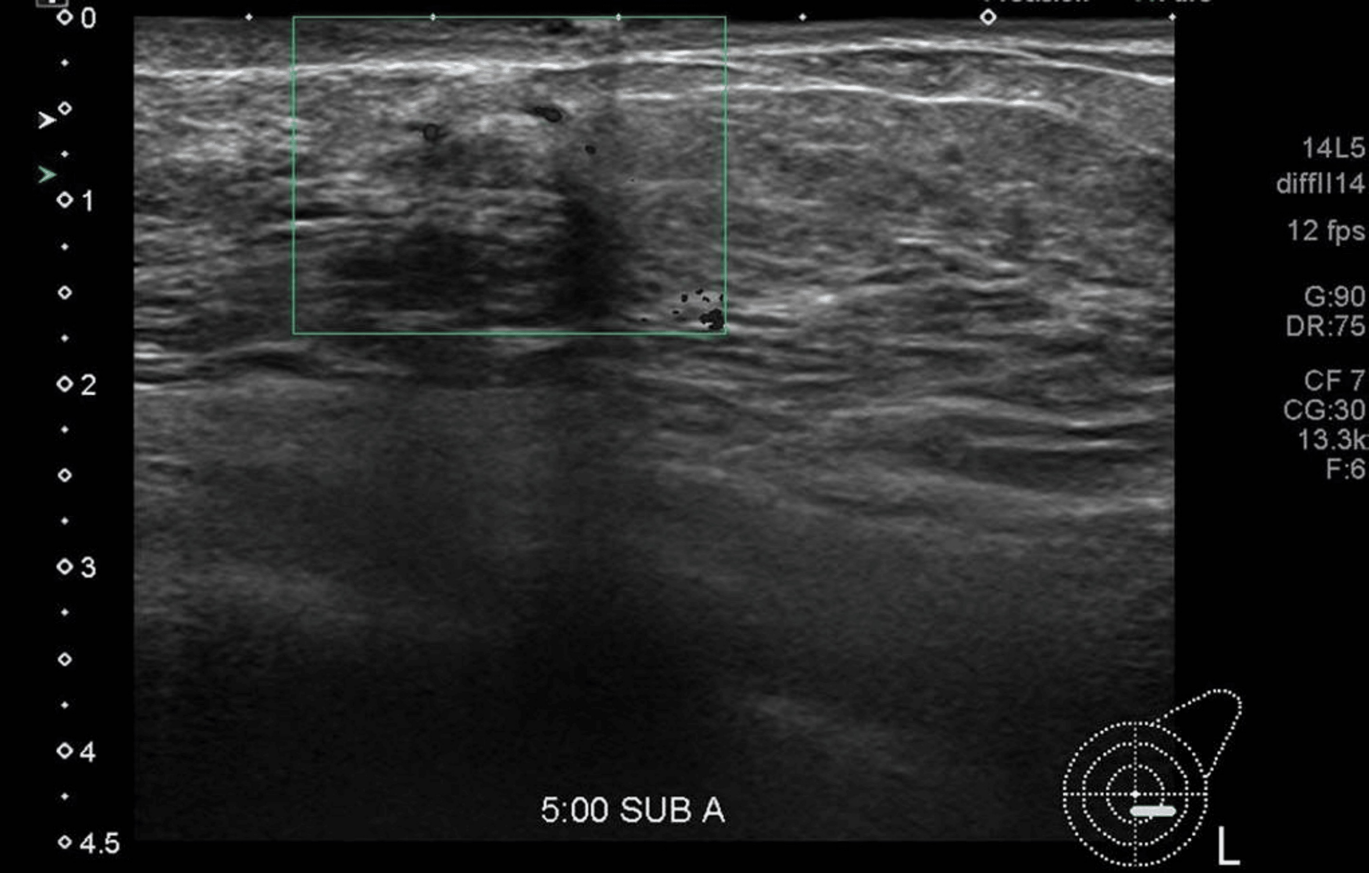
Follow-up imaging Follow-up ultrasound imaging more than two years post-procedure showed a faintly appreciable, heterogeneous, subcentimeter focus at the cryoablation site, with no new suspicious lesions detected in the left breast.

Follow-up imaging at six months and at one, two, and three years revealed progressive reduction in lesion size. The tumor gradually evolved into a subcentimeter heterogeneous scar-like region (Figure 6). At the most recent follow-up, approximately 32 months post-ablation, the lesion measured 0.7 cm and appeared less distinct on ultrasound. During follow-up, there was no evidence of recurrence in either breast, axillae, or distant sites, confirmed through clinical examination, serial ultrasound, mammography, and tumor marker assessment. Serial tumor markers remained within normal limits throughout the surveillance period (e.g., carbohydrate antigen (CA) 15-3: 12.1 U/mL; carcinoembryonic antigen (CEA): 3.99 µg/L). The patient reported resolution of the palpable abnormality within the first year, remained asymptomatic, and expressed satisfaction with her outcome thus far.

Clinical breast examination showed no signs of recurrence, lymphedema, or metastases. The patient adhered well to tamoxifen and lifestyle advice. She is planned for routine follow-up with annual imaging. Given her age, frailty, tumor biology, and procedural success, this case demonstrates the feasibility, safety, and intermediate-term efficacy of cryoablation as an alternative to surgery for elderly patients with superficial, hormone-positive breast cancer.

## Discussion

This case underscores the potential of cryoablation as a curative alternative to surgery for select elderly patients with early-stage breast cancer, even when tumors abut the dermis. Superficial lesions are conventionally excluded due to the risk of skin injury; however, when combined with real-time hydrodissection, ablation can be performed safely [6,10]. In our case, rather than relying on predetermined fluid volumes, we maintained continuous displacement of the skin away from the tumor.

Cryoablation is generally well tolerated, with most adverse events reported as minor (88.2%) or moderate (9.6%), including pain, ecchymosis, fat necrosis, seroma, or infection, and no serious device-related complications when performed under appropriate precautions [2,3,7]. In superficially located lesions, skin frostbite and necrosis remain important risks, which can be effectively mitigated by continuous hydrodissection, as demonstrated here.

The outcome of sustained tumor control approaching three years, along with high patient satisfaction, is consistent with findings from larger studies such as ICE3 [7]. In the five-year ICE3 analysis, the ipsilateral breast tumor recurrence rate was 4.3%, with breast cancer-specific survival of 96.7% and 100% physician and patient satisfaction with cosmetic results [7]. They also support findings from pilot series showing cryoablation is safe, effective, and well tolerated in small invasive tumors under local anesthesia [1,12,13]. In particular, prior work has shown complete ablation in tumors <1.5 cm in size and limited ductal carcinoma in situ component [14].

Invasive mucinous carcinoma rarely features lymph node involvement and generally has a good prognosis [15]. Given its favorable biology, characterized by indolent growth, low histological grade, and infrequent lymph node involvement, this histologic subtype may be particularly amenable to minimally invasive approaches in patients with comorbidities or high operative risk [16].

The technique described here extends the applicability of cryoablation by demonstrating technical feasibility and oncologic effectiveness in superficially located lesions, an area previously underrepresented in literature.

This case also highlights the value of collaborative decision-making in the care of older adults with cancer, where achieving cancer control must be balanced with honoring patient autonomy, preserving quality of life, and minimizing the risks associated with surgery [17]. As imaging-guided ablation techniques evolve and evidence accumulates, cryoablation may become a viable primary treatment for carefully selected elderly patients.

## Conclusions

Breast cryoablation is a developing treatment option in Singapore. To our knowledge, this is the first case reported to show intermediate-term success in managing a superficial invasive mucinous carcinoma located close to the skin. The consistent clinical and imaging findings over nearly three years, along with the patient’s positive experience, support the use of cryoablation as a safe and effective treatment for selected individuals who are not suitable for surgery. This case demonstrates how cryoablation can be adapted to treat tumors near the skin by using real-time hydrodissection to protect surrounding tissue. It also highlights the importance of tailoring breast cancer treatment to the needs of older adults. As research in this area continues to grow, this case supports wider consideration of image-guided treatments for breast cancer, especially in low-risk tumors and in situations where preserving quality of life and respecting patient preferences are important considerations.
